# Exploring new horizons in mid-to-far infrared nonlinear optical crystals: the significant potential of trigonal pyramidal [TeS_3_]^2−^ functional units†

**DOI:** 10.1039/d4sc07322c

**Published:** 2025-01-10

**Authors:** Bo Zhang, Sheng-Hua Zhou, Bing-Xuan Li, Xin-Tao Wu, Hua Lin, Qi-Long Zhu

**Affiliations:** a State Key Laboratory of Structural Chemistry, Fujian Institute of Research on the Structure of Matter, Chinese Academy of Sciences Fuzhou 350002 China linhua@fjirsm.ac.cn qlzhu@fjirsm.ac.cn; b Fujian Science & Technology Innovation Laboratory for Optoelectronic Information of China Fuzhou 350002 China; c University of the Chinese Academy of Sciences Beijing 100049 China; d Resource Environment & Clean Energy Laboratory, School of Chemistry and Chemical Engineering, Jiangsu University of Technology Changzhou 213001 China zhoushenghua@jsut.edu.cn

## Abstract

Traditional tetrahedral-based mid-to-far infrared (MFIR) nonlinear optical (NLO) crystals often face limitations due to the optical anisotropy constraints imposed by their highly symmetric structures. In contrast, the relatively rare trigonal pyramidal [TeS_3_]^2−^ functional unit characterized by its asymmetric structure and stereochemically active lone pair (SCALP), offers improved optical anisotropy, hyperpolarizability and a broader IR transparency range. Despite its potential, synthetic challenges have hindered the development of MFIR NLO crystals that incorporate this unit, with only one example reported to date. Herein, an innovative MFIR NLO crystal, Cu_10_Te_4_S_13_ has been successfully constructed using the trigonal pyramidal [TeS_3_]^2−^ units, *via* a simple high-temperature solid-state method. The novel three-dimensional structure of Cu_10_Te_4_S_13_ is interconnected by butterfly-orchid-like [Cu_6_Te_4_S_13_] anionic clusters and [CuS_4_] groups, where the former are composed of trigonal pyramidal [TeS_3_]^2−^ groups and [Cu_6_S_13_] hexamers. Cu_10_Te_4_S_13_ exhibits a remarkable second harmonic generation effect, approximately 3.75 times that of AgGaS_2_ at 2900 nm in the particle size range of 30–45 μm. Additionally, it demonstrates favorable crystal growth habits, producing single crystals with maximum dimensions of about 7 × 3 × 2 mm^3^. This polished single crystal appears to exhibit complete transparency within the MFIR spectral window ranging from 2.5 to 25 μm, representing the widest IR transmission in all reported NLO chalcogenides. Furthermore, the structure–property relationship is also elucidated through first-principles analysis. This work confirms the potential of the unique trigonal pyramidal [TeS_3_]^2−^ as a MFIR NLO functional unit, paving the way for the development of unconventional MFIR NLO materials.

## Abstract 2

The [TeS_3_]^2−^ unit with a SCALP disrupts tetrahedral symmetry, leading to the synthesis of Cu_10_Te_4_S_13_ for the first time. This material exhibits strong SHG (3.75 × AgGaS_2_) and the widest MFIR transparency (2.5–25 μm) reported for NLO chalcogenides.

## Graphical abstract

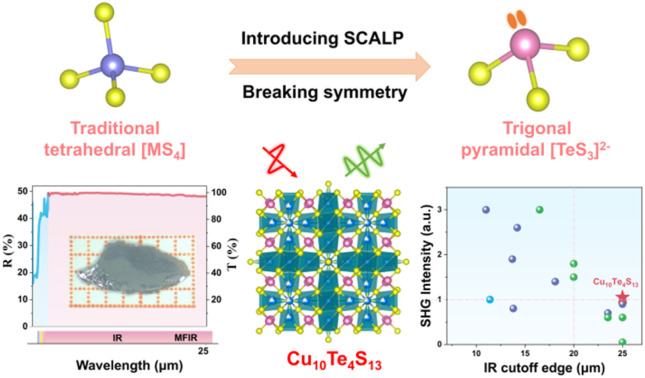

## Introduction

Mid-to-far infrared (MFIR) nonlinear optical (NLO) crystals are essential for advancements in IR technology, significantly improving the performance, stability, and application range of IR light sources.^1^ As the demand for IR technology increases across various sectors, including the military, aerospace, environmental monitoring, and communications, the quest for high-performance MFIR NLO crystals has become increasingly paramount.^2^ Currently, the availability of effective MFIR NLO crystals, particularly those operating beyond 5 μm, is limited. Traditional MFIR NLO crystals, such as chalcopyrite-type AgGaS_2_,^3^ AgGaSe_2_,^4^ and ZnGeP_2_,^5^ face significant challenges due to their inherent limitations. These materials often exhibit intrinsic defects that severely restrict their high-power laser output. Moreover, AgGaS_2_ and ZnGeP_2_ demonstrate pronounced multi-phonon absorption at around 9 μm, which prevents them from effectively utilizing the vital “8–14 μm” transparency window.^6,7^ Given these constraints, the urgent development of novel MFIR NLO candidates with enhanced properties is not only desirable but essential.

Traditional tetrahedral-based MFIR NLO crystals often face limitations due to the optical anisotropy constraints imposed by their highly symmetric structures.^8–21^ To enhance optical anisotropy, it is imperative to explore other MFIR NLO functional units beyond tetrahedral units. Compared to tetrahedral units, asymmetric functional units with stereochemically active lone pairs (SCALPs) have attracted our attention due to their enhanced optical anisotropy and improved hyperpolarizability.^22–28^ Among these, many triangular pyramidal functional units exhibit broader IR transmission than their tetrahedral counterparts, making them more suitable for practical applications in MFIR NLO crystals.^29^ Additionally, trigonal pyramids offer greater structural flexibility and design potential than tetrahedral motifs. This design flexibility permits researchers to explore and optimize the overall performance of NLO materials to meet specific requirements.

Currently, NLO crystals based on trigonal pyramidal functional units have been more extensively studied in the ultraviolet-visible (UV-vis) range compared to the MFIR range. Among them, the [TeO_3_]^2−^ group has been recognized as an excellent UV-vis NLO-active functional unit.^30–38^ Over the past two decades, extensive research has focused on NLO crystals containing [TeO_3_]^2−^ units, leading to the discovery of many NLO materials with high optical anisotropy and strong second-harmonic generation (SHG) effects, such as Cd_2_Nb_2_Te_4_O_15_ (31 × KDP, 0.12 @ 546 nm),^35^ Mo(H_2_O)Te_2_O_7_ (5.4 × KDP, 0.528 @ 546 nm),^36^ β-K_2_TeW_3_O_12_ (15 × KDP, 0.196 @ 1064 nm),^37^ and LiNbTeO_5_ (17 × KDP, 0.083 @ 632.8 nm).^38^ The [TeO_3_]^2−^ unit offers several advantages for constructing NLO materials: (i) its highly asymmetric coordination environments facilitate the formation of non-centrosymmetric (NCS) structures;^39^ (ii) the uniform arrangement of asymmetric trigonal pyramidal units enhances optical anisotropy, conducive to a large birefringence;^40^ (iii) SCALPs in Te^4+^ promote secondary Jahn–Teller distortions, favoring powerful SHG effects.^35,41^ Nevertheless, the strong absorption of Te–O bonds in the 400–800 cm^−1^ IR range prevents their application in the MFIR range.^42^ To overcome this limitation, the oxygen atoms in the [TeO_3_]^2−^ unit are replaced with sulfur to derive the pyramidal [TeS_3_]^2−^ units. [TeS_3_]^2−^ not only retains the trigonal pyramidal configuration with a pair of SCALPs, inheriting the advantages of large optical anisotropy and hyperpolarizability of [TeO_3_]^2−^ units, but also extends its transmission window to the MFIR range. Consequently, [TeS_3_]^2−^ presents itself as a promising candidate for MFIR NLO applications, distinct from traditional tetrahedral functional units.

Nonetheless, only one NLO crystal containing the [TeS_3_]^2−^ unit, CsAg_2_TeS_6_,^43^ has been reported. This limited occurrence is primarily due to synthetic challenges: (i) bond strength: Te–S bonds are relatively weak compared to Te–O bonds. Additionally, the small electronegativity difference between Te and S complicates the formation and stability of the [TeS_3_]^2−^ units during synthesis. (ii) Chemical properties of Te: as a metalloid, Te displays complex chemical behavior, exhibiting multiple oxidation states with varying reactivities and reaction products. This variability requires precise control of synthesis conditions to ensure that Te remains in the desired oxidation state, further complicating the process of creating compounds with [TeS_3_]^2−^ units. As a result, research on NLO crystals containing [TeS_3_]^2−^ units is greatly limited.

Cu^+^ with a d^10^ electronic configuration exhibits a large polar displacement, which contributes to strong SHG effects.^44^ Additionally, Cu^+^ can adopt multiple coordination modes, namely CuS_*n*_ (*n* = 2–4), further enhancing the structural diversity of compounds.^45–47^ Utilizing [TeS_3_]^2−^ as the functional units and Cu^+^ as the cations, we successfully synthesized a novel MFIR NLO crystal, Cu_10_Te_4_S_13_ through a high-temperature solid-state reaction. It exhibits a remarkable SHG effect, measuring 3.75 times that of AgGaS_2_ at 2900 nm. Furthermore, Cu_10_Te_4_S_13_ demonstrates favorable crystallographic growth habits, producing single crystals with dimensions up to 7 × 3 × 2 mm^3^. Such polished single crystals display a complete transmission window across 2.5–25 μm, representing the widest IR transmission in all reported NLO chalcogenides. First-principles analysis elucidates the NLO and linear optical properties, providing insights into the structure–property relationship. Our research confirms the promise of the [TeS_3_]^2−^ unit as an MFIR NLO-active functional unit and presents a novel perspective for designing and exploring MFIR NLO materials beyond traditional chalcopyrite structures.

## Results and discussion

Using the high-temperature solid-state method, black metallic lustrous block Cu_10_Te_4_S_13_ single crystals were successfully synthesized. Energy-dispersive X-ray spectroscopy (EDS) elemental analysis revealed the presence of Cu, Te, and S elements in the single crystals (Fig. S1, ESI†), and Cu : Te : S = 9.7 : 4 : 12.4, close to the atomic ratio of the molecular formula. Field emission scanning electron microscopy (FESEM) analysis confirmed the uniform distribution of Cu, Te, and S elements (Fig. S2, ESI†).

**Fig. 1:**
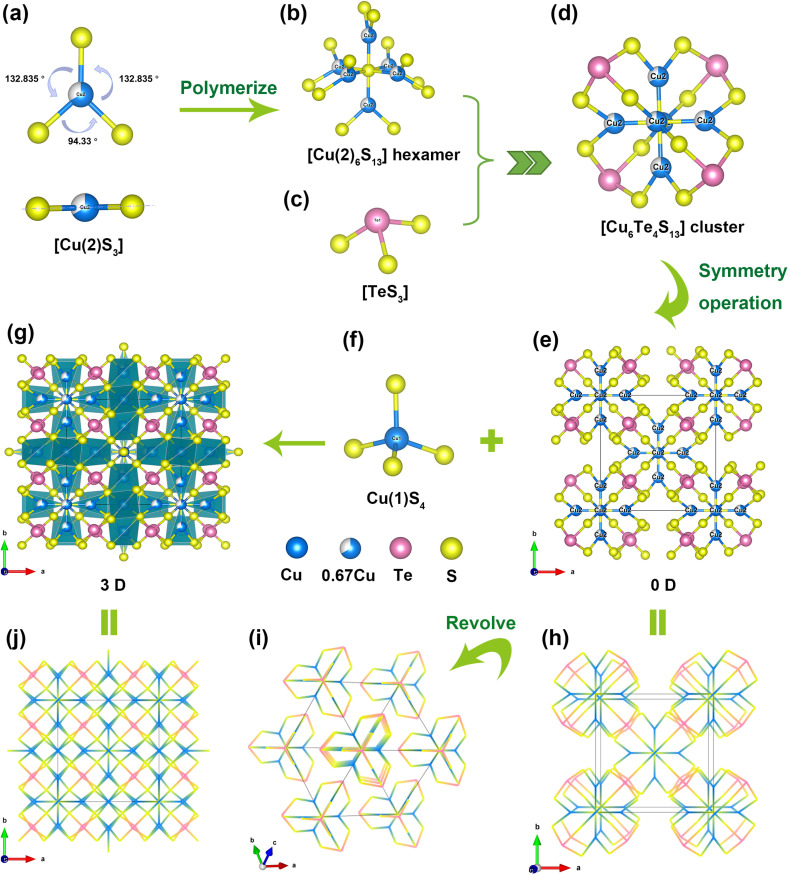
(a) Planar triangular [Cu(2)S_3_] motif; (b) unique [Cu_6_S_13_] hexamer; (c) [TeS_3_] triangular pyramid; (d) butterfly-orchid-like [Cu_6_Te_4_S_13_]^4−^ 0D anion cluster; (e) [Cu(2)–Te–S] 0D framework; (f) [Cu(1)S_4_] tetrahedron; (g) the 3D structure of Cu_10_Te_4_S_13_; (h and i) the wireframe of the 0D framework; (j) the wireframe of the 3D structure.

The crystal structure of Cu_10_Te_4_S_13_ was determined by single-crystal X-ray diffraction, with crystallographic data summarized in Table S1.† Cu_10_Te_4_S_13_ crystallizes in the NCS space group *I*4̄3*m* [Pearson code: CI58; Wyckoff sequence: gedca]. Within its asymmetric unit, there are two unique Cu, one unique Te and two unique S atoms (Table S2†). The Cu(2) atoms adopt three-coordinated planar triangular [Cu(2)S_3_] motifs, where Cu(2)–S bond lengths range from 2.2173(18) to 2.183(2) Å, and S–Cu(2)–S bond angles vary from 94.33(2) to 132.83(6)°. Moreover, the sum of the three bond angles around Cu(2) is 360° (Fig. 1a). The Cu(2) atom is located at the central position of the triangular plane determined by the three S atoms (Fig. 1a), and these results all confirm that the [CuS_3_] motif is planar triangular. In the structure of Cu_10_Te_4_S_13_, six planar [Cu(2)S_3_] units share the S(2) atom located at the Wyckoff 2a position to form a unique [Cu(2)_6_S_13_] hexamer (Fig. 1b). As depicted in Fig. 1c, Te(iv) atoms exhibit triangular pyramid coordination with three S atoms forming [TeS_3_] motifs, with Te–S bond lengths of 2.3812 Å. Four [TeS_3_] units are connected to the hexamer by sharing the S(1) atoms of the [Cu(2)_6_S_13_] hexamer, forming a butterfly-orchid-like [Cu_6_Te_4_S_13_]^4−^ zero-dimensional (0D) anion cluster (Fig. 1d). The anionic clusters stack repetitively along the *a*-, *b*-, and *c*-axes by symmetry operation, constituting a [Cu(2)–Te–S] 0D anionic framework, with the centers of the clusters residing at the body-centered and vertex positions within the unit cell (Fig. 1e, h and i).

Two types of S atoms respectively form [S(1)Cu_3_Te] tetrahedral and [S(2)Cu_6_] octahedral groups (Fig. S3†). Each Cu(1) atom is coordinated by four S atoms forming a [CuS_4_] tetrahedral unit (Fig. 1f), with Cu(1)–S bond lengths ranging from 2.3233(7) to 2.3233(8) Å. Notably, the Cu(1) atom located at the Wyckoff 12d position is independently occupied in crystallography, while the Cu(2) atom at the Wyckoff 12e position is partially occupied in crystallography, with a 2/3 occupancy and a 1/3 vacancy occupancy in a single cell. This partial occupancy of Cu is a common feature in many Cu-containing structures, such as CuZnPS_4_ (ref. 45) and Cu_5_Zn_0.5_P_2_S_8_.^46^ The 0D anionic framework is interconnected by the [Cu(1)S_4_] units with tetrahedral configurations, resulting in the ultimate three-dimensional (3D) structure of Cu_10_Te_4_S_13_ (Fig. 1g and j). In conclusion, Cu_10_Te_4_S_13_ is an interesting and unique 3D structure composed of three completely different configurations of functional units, namely the trigonal pyramid configuration [TeS_3_], the planar triangle [CuS_3_] group and the tetrahedral configuration [CuS_4_] unit, which are interconnected by sharing S atoms.

Additionally, the phenomenon of multiple coordination modes of metal centers is commonly observed in chalcogenides containing d^10^ metals.^48^ The crystal structure can be quantitatively evaluated through bond valence sum (BVS) and global instability index (*G*) calculations. As shown in Table S3,† BVS calculations for Cu(1), Cu(2), Te, S(1) and S(2) yield 1.1432, 1.1789, 3.6129, 1.1968 and 2.5050 respectively, indicating oxidation states of +1, +1, +4, −2 and −2. The calculated BVS values can be used with the expected bond valence (*v*_*i*_) of each ion for *G* calculation (eqn (1)).1
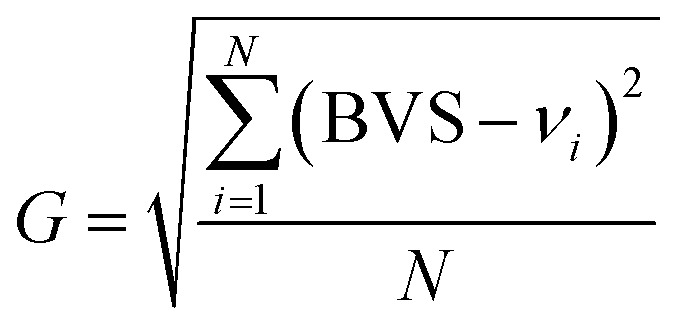
where *N* is the number of atoms within the formula unit.^49^ The derived *G*-value reflects the strain level within the crystal structure, with values less than 0.05 indicating minimal strain—a result anticipated due to experimental uncertainties. A *G*-value ranging from 0.05 to 0.20 suggests a crystal structure subjected to moderate and reasonable strain, while values exceeding 0.20 denote significant strain indicative of potential instability in the crystalline structure. As computationally determined, the *G*-value for Cu_10_Te_4_S_13_ stands at 0.20, signifying a rational degree of spatial strain. This is comparable to reported *G*-values for compounds containing Cu^+^, such as Cu_4_ZnGe_2_S_7_ (*G* = 0.20),^50^ Cu_4_CdGe_2_S_7_ (*G* = 0.19),^50^ and Cu_2_MnGeS_4_ (*G* = 0.18).^51^ Tables S3 and S4† provide detailed information on the bond lengths, bond valences, and bond angles for Cu_10_Te_4_S_13_.

The calculation results of Mulliken population (MP) for bonds and atoms in Cu_10_Te_4_S_13_ and AgGaS_2_ are shown in Table S5.†^52^ In Cu_10_Te_4_S_13_, the MP of the Cu(1)–S(1) bond in the [CuS_4_] building block is 0.38, resulting in an average MP of 0.38 for the [CuS_4_] motif. In the planar π-conjugated [CuS_3_] building block, the MPs of the two types of Cu–S bonds are 0.30 and 0.56, with an average MP of 0.39. For AgGaS_2_, the average MP of the Ag–S bond is 0.29, which is smaller than that of the Cu–S bond in Cu_10_Te_4_S_13_ (0.38–0.39). This confirms that the covalence of the Cu–S bonds in Cu_10_Te_4_S_13_ is greater than that of the Ag–S bonds in AgGaS_2_, and the overall covalence of the planar π-conjugated [CuS_3_] motif is greater than that of the tetrahedral [CuS_4_] motif. Furthermore, the MP value for Te–S bonds in Cu_10_Te_4_S_13_ is 0.32, which is smaller than the 0.42 for Ga–S bonds in AgGaS_2_, aligning with established trends. Notably, the charge on Te atoms in Cu_10_Te_4_S_13_ is 0.86*e*, greater than that of Ga atoms in AgGaS_2_ (0.82*e*), indicating that Te indeed carries a higher charge than Ga. This finding concurs with bond valence calculations, wherein Te adopts a +4 oxidation state while Ga exhibits a +3 state.

**Fig. 2:**
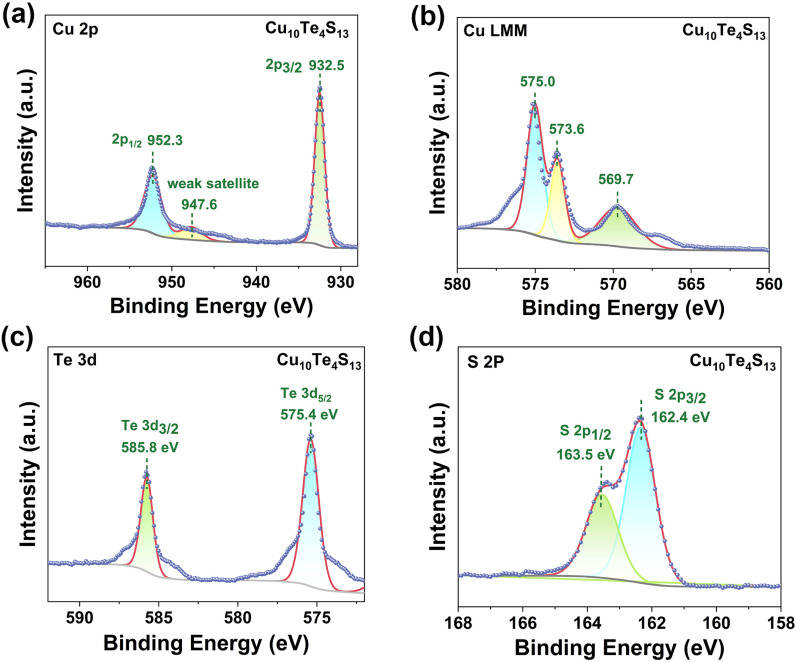
(a) Cu 2p XP spectrum; (b) Cu LMM auger XP spectrum; (c) Te 3d XP spectrum; (d) S 2p XP spectrum of Cu_10_Te_4_S_13_.

The valence states of each element in Cu_10_Te_4_S_13_ were probed by X-ray photoelectron spectroscopy (XPS). The binding energy (BE) for surface charging was calibrated by taking the C 1s peak of contaminated carbon as a reference at 284.5 eV. The Cu 2p XP spectrum exhibits two main peaks at 932.5 eV and 952.3 eV, respectively, with a splitting (Δ) value of 19.8 eV, corresponding to Cu 2p_1/2_ and 2p_3/2_.^53^ Additionally, besides the two main peaks, there is an extremely weak satellite peak at around 947.6 eV, indicating the presence of Cu^+^ in the sample (Fig. 2a).^54^ Furthermore, we confirmed the existence of Cu^+^ at BE ∼569.7 eV through the Auger Cu LMM spectrum (Fig. 2b), which is in accordance with the previous reports on Cu-containing compounds.^55^ In the Te 3d XP spectrum, two main peaks were detected (575.4 and 585.8 eV), corresponding to Te^4+^ 3d_5/2_ and 3d_3/2_, confirming that the valence of Te in the sample is +4 (Fig. 2c). As depicted in Fig. 2d, the BEs of S 2p_3/2_ and S 2p_1/2_ are 162.4 eV and 163.5 eV, respectively, and the Δ value is 1.1 eV, which is typically attributed to S^2−^.

**Fig. 3:**
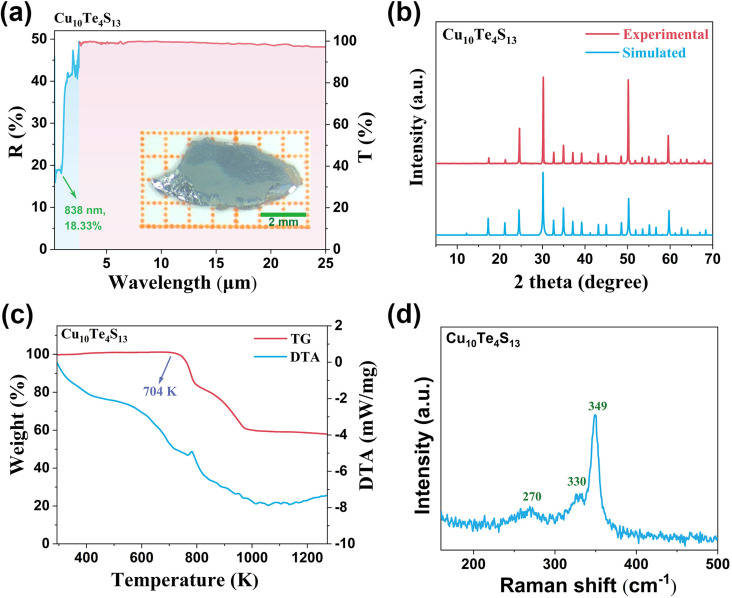
(a) The UV-vis-IR spectrum of Cu_10_Te_4_S_13_ and the inset shows a photo of a large polished crystal of Cu_10_Te_4_S_13_; (b) simulated and experimental powder X-ray diffraction patterns of Cu_10_Te_4_S_13_; (c) TG-DTA of Cu_10_Te_4_S_13_ under a N_2_ atmosphere; (d) Raman spectrum of Cu_10_Te_4_S_13_.

The UV-vis-NIR diffuse reflectance spectrum of Cu_10_Te_4_S_13_ was measured in the range of 200–2500 nm, and its bandgap was inferred to be 1.09 eV (Fig. S4†). As depicted in Fig. 3a, Cu_10_Te_4_S_13_ is transparent from 0.84 to 25 μm, spanning visible and MFIR spectral regions. The IR transmission spectrum of Cu_10_Te_4_S_13_ was tested using a single crystal after polishing. It exhibits complete transmission in the range of 2.5–25 μm, encompassing the vital MFIR atmospheric windows, and can be effectively simulated by laser sources at 1.064, 2.09, and 10.6 μm wavelengths. As shown in Fig. 3b, experimental powder X-ray diffraction (PXRD) patterns of Cu_10_Te_4_S_13_ matched well with calculated results, confirming the purity.

The thermogravimetric-differential thermal analysis (TG-DTA) of Cu_10_Te_4_S_13_ was conducted in a nitrogen atmosphere within the temperature range of 293–1273 K. As shown in Fig. 3c, Cu_10_Te_4_S_13_ can remain stable at 704 K. Such thermal stability is higher than that of some NLO crystals featuring pyramidal motifs, such as Li_2_TeSe_3_ (701 K),^56^ Hg_3_AsS_4_Cl (578 K),^16^ and Hg_3_AsS_4_Br (603 K).^16^

The Raman spectrum of Cu_10_Te_4_S_13_, spanning the range from 500 to 100 cm^−1^, is presented in Fig. 3d. The [TeS_3_] units within Cu_10_Te_4_S_13_ exhibit an ideal *C*_3V_ symmetry, a crystalline feature that manifests in the Raman spectrum as precisely two distinctive vibrational bands. One of these bands is attributed to the *ν*_asym_ mode at 359 cm^−1^, while the other, positioned at 336 cm^−1^, corresponds to the *ν*_sym_ mode of the Te–S bond. Additionally, the peak at 270 cm^−1^ is identified as arising from Cu–S vibrational activity.^57^

**Fig. 4:**
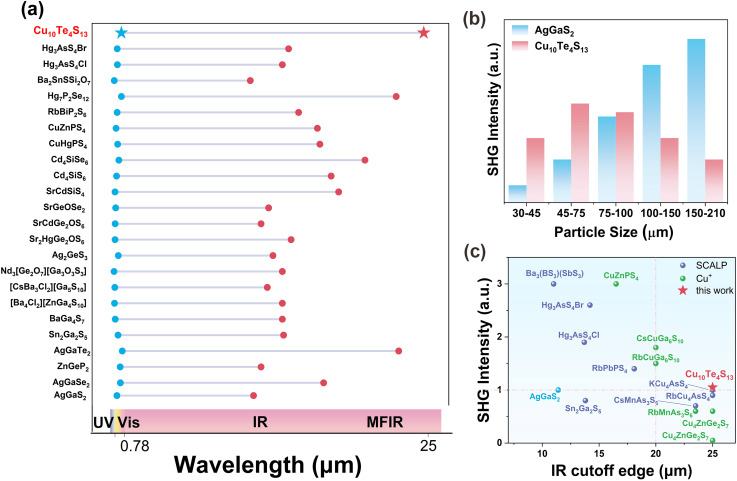
(a) A comparison of the transparency range between Cu_10_Te_4_S_13_ and some typical IR NLO materials; (b) SHG intensity *versus* particle size curves at 2900 nm for Cu_10_Te_4_S_13_ and AgGaS_2_; (c) a statistical comparison of the SHG effect (*ca.* 70–110 μm) and IR cutoff edge between Cu_10_Te_4_S_13_ (red star) and several typical IR NLO materials, including those with SCALP units (purple sphere) or containing Cu^+^ (green sphere), with AgGaS_2_ (blue sphere) as the reference.

As shown in Table S6† and Fig. 4a, the low-energy end corresponding to the transmission range of Cu_10_Te_4_S_13_ is longer than that of current excellent IR NLO crystals, such as Hg_3_AsS_4_Cl (13.7 μm),^16^ Hg_3_AsS_4_Br (14.2 μm),^16^ AgGaS_2_ (11.4 μm),^58^ Ag_2_GeS_3_ (12.95 μm),^59^ Hg_7_P_2_Se_12_ (22.8 μm),^7^ AgGaTe_2_ (23 μm),^41,60^*etc.* This indicates that Cu_10_Te_4_S_13_ is a promising candidate material for MFIR NLO crystal applications.

Employing the Kurtz–Perry method, the powder SHG intensities of Cu_10_Te_4_S_13_ and AgGaS_2_ were measured under Q-switched laser irradiation at 2900 nm. As shown in Fig. 4b, Cu_10_Te_4_S_13_ achieves the maximum SHG intensity (3.75 × AgGaS_2_) in the particle size range of 30–45 μm. Its SHG intensity is greater than that of many NLO crystals characterized by pyramidal motifs, such as Na_4_SrAs_2_S_8_ (0.95 × AgGaS_2_),^61^ KAg_2_AsS_4_ (1.36 × AgGaS_2_),^62^ RbAg_2_AsS_4_ (1.34 × AgGaS_2_),^62^ and Ag_3_AsS_3_ (1.1 × AgGaS_2_).^63^ Generally, chalcogenides containing SCALP groups tend to exhibit greater structural anisotropy, leading to higher birefringence (Δ*n*) and enabling phase matching in the infrared range.^64^ However, despite containing SCALP groups, Cu_10_Te_4_S_13_ cannot achieve phase matching at 2900 nm due to its cubic crystal system (Fig. 4b), which results in an isotropic structure (Δ*n* = 0). In future work, a chemical substitution strategy^65^ could be considered, where alkali or alkaline earth metals with high electronegativity replace the Cu element. This would break the high symmetry of the structure and potentially enhance Δ*n*, thereby enabling phase matching. A statistical analysis and comparison of the SHG intensities (70–110 μm) and IR cutoff edge of Cu_10_Te_4_S_13_ and some currently reported typical IR NLO crystal materials characterized by triangular pyramidal units were carried out. The results show that Cu_10_Te_4_S_13_ has good balanced properties: a long IR cutoff edge greater than 25 μm and moderate SHG effect of 1.05 × AgGaS_2_ in the particle size range of 70–100 μm (Fig. 4c and Table S7†). This shows that the novel [TeS_3_]^2−^ group with a pair of SCALPs is a potential MFIR NLO active motif.

**Fig. 5:**
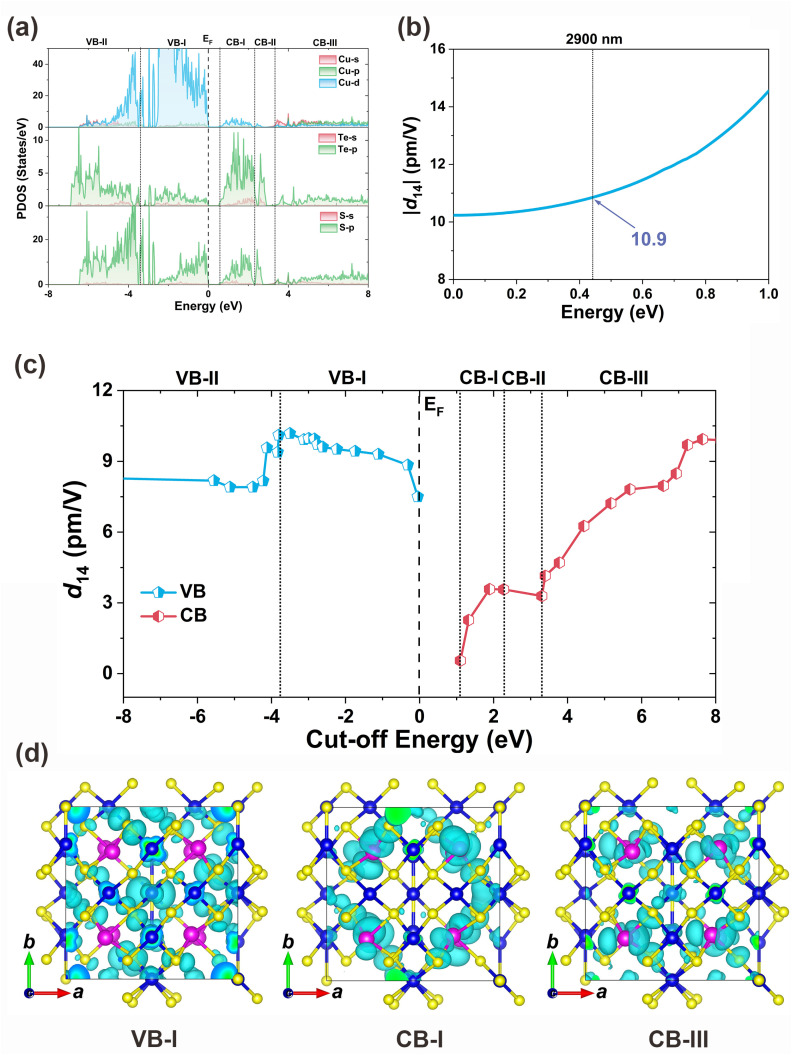
Theoretical calculations of Cu_10_Te_4_S_13_: (a) PDOS diagrams projected onto constituent atoms; (b) frequency-dependent SHG tensor *d*_14_; (c) relationship between SHG tensor *d*_14_ (pm V^−1^) and cut-off energy (eV); (d) partial charge density distribution diagrams showing the main contributions of VB-I, CB-I and CB-III regions. Blue atoms represent Cu, pink atoms represent Te, and yellow atoms represent S.

To elucidate the intrinsic structure–property relationship of Cu_10_Te_4_S_13_, we conducted a systematic first-principles computational study.^66^ Cu_10_Te_4_S_13_ exhibits a direct band gap of 0.82 eV based on PBE calculations. When accounting for the spin–orbit coupling effect of Te^4+^, the theoretical band gap decreases to 0.67 eV. For a more accurate estimation, HSE calculations were also performed, yielding a theoretical band gap of 1.42 eV (see Fig. S5† for details). Analyzing the partial density of states (PDOS) diagram can reveal the origin of optical properties. In the PDOS diagram of Cu_10_Te_4_S_13_, the top of the valence band (VB) is mainly composed of Cu 3d and S 3p orbitals, while the bottom of the conduction band (CB) is mainly contributed by Te 5p and S 3p orbitals (Fig. 5a). Therefore, the optical properties of Cu_10_Te_4_S_13_ are mainly synergistically determined by the [CuS_3_], [CuS_4_], and [TeS_3_] FBUs. Considering the *I*4̄3*m* point group and Kleinman symmetry rules, Cu_10_Te_4_S_13_ has only one independent non-zero tensor (*d*_14_). At a wavelength of 2900 nm, the SHG tensor is calculated to be *d*_14_ = 10.9 pm V^−1^ (Fig. 5b), which is approximately 0.8 times more than that of *d*_36_ = 13.4 pm V^−1^ of AgGaS_2_. It should be noted that the calculated SHG value is smaller than the measured one, and this discrepancy may arise from two main factors. First, the Cu(2) sites in the actual structure are partially occupied, so we used an approximate structure in our calculations, which could lead to deviations. Second, the theoretical model assumes a perfect crystal and does not account for factors such as particle size, whereas in practice, the SHG intensity is influenced by crystal quality and particle size. For example, the SHG intensity for samples with particle sizes in the range of 74–106 μm is closer to the theoretical value. In addition, a cut-off energy dependence analysis of the SHG coefficient *d*_14_ is performed to clarify the contribution of orbitals.^67^ We find that its SHG effect mainly comes from the VB-I, CB-I, and CB-III regions (Fig. 5c). Through the corresponding analysis of PDOS and charge density diagram (Fig. 5d), it is found that its SHG effect is mainly contributed by the Cu 3d, S 3p, and Te 5p orbitals corresponding to the triangular [CuS_3_] (19.5%), tetrahedral [CuS_4_] (19.6%), and triangular pyramid [TeS_3_] (60.9%) units.

## Conclusions

In summary, Cu_10_Te_4_S_13_, a novel crystal containing a trigonal pyramidal [TeS_3_]^2−^ MFIR NLO-active functional unit, has been synthesized for the first time. It exhibits favorable crystal growth habits (7 × 3 × 2 mm^3^) and remarkable SHG efficiency (3.75 × AgGaS_2_ at 2900 nm). Moreover, single crystal IR transmission tests confirm that it is completely transparent across the 2.5–25 μm range. These findings indicate that Cu_10_Te_4_S_13_ is a promising MFIR NLO candidate material and confirm for the first time that the [TeS_3_]^2−^ motif represents a potential MFIR NLO functional unit. The incorporation of the [TeS_3_]^2−^ functional units, or analogous triangular pyramidal units, into future materials research may well spawn a novel class of MFIR NLO materials, poised for practical utilization, fundamentally distinct from the conventional chalcopyrite-type MFIR materials in terms of their unique structural configurations. In essence, this study opens up exciting avenues for the exploration and development of innovative MFIR NLO materials.

## Data availability

All supplementary data for the results of this study are available in the article and its ESI file.† Crystallographic data for Cu_10_Te_4_S_13_ have been deposited at the CCDC under 2391396 and can be obtained from https://www.ccdc.cam.ac.uk/structures.

## Author contributions

Bo Zhang: investigation, formal analysis, writing – original draft. Sheng-Hua Zhou: investigation, methodology, validation. Bing-Xuan Li: investigation, formal analysis. Xin-Tao Wu: conceptualization, writing – review & editing. Hua Lin: supervision, conceptualization, writing – review & editing. Qi-Long Zhu: supervision, writing – review & editing.

## Conflicts of interest

There are no conflicts to declare.

## Supplementary Material

SC-OLF-D4SC07322C-s001

SC-OLF-D4SC07322C-s002
