# Supplementary material for: Exploring new horizons in mid-to-far infrared nonlinear optical crystals: the significant potential of trigonal pyramidal [TeS3]2− functional units
Source: Chem Sci. 2025 Jan 10;16(7):3218–27. doi: 10.1039/d4sc07322c (PMC11744484; doi:10.1039/d4sc07322c)
Supplement: SC-016-D4SC07322C-s001 [file SC-016-D4SC07322C-s001.pdf]

Supplementary Information (SI) for Chemical Science.
This journal is © The Royal Society of Chemistry 2025

Electronic Supplementary Information (ESI)

Exploring New Horizons in Mid-to-Far Infrared Nonlinear Optical Crystals: The Significant Potential of Trigonal Pyramidal $[TeS_3]^{2-}$ Functional Units

Bo Zhang,[a,b,c] Sheng-Hua Zhou,[*,a,d] Bing-Xuan Li,[a,b] Xin-Tao Wu,[a,b] Hua Lin[*,a,b] and Qi-Long Zhu[*,a,b]

[a]*State Key Laboratory of Structural Chemistry, Fujian Institute of Research on the Structure of Matter, Chinese Academy of Sciences, Fuzhou 350002, China*

[b]*Fujian Science & Technology Innovation Laboratory for Optoelectronic Information of China, Fuzhou, Fujian 350108, China*

[c]*University of Chinese Academy of Sciences, Beijing 100049, China*

[d]*Resource environment & Clean Energy Laboratory, School of Chemistry and Chemical Engineering, Jiangsu University of Technology, Changzhou 213001, China*

*E-mail: linhua@fjirsm.ac.cn, qlzhu@fjirsm.ac.cn, and zhoushenghua@jsut.edu.cn.

Electronic Supplementary Information (ESI)

Contents

Experimental Section 3

Computational Method..4

Figure S1. The energy-dispersive X-ray spectroscopy for $Cu_{10}Te_4S_{13}$. 7

Figure S2. SEM images of $Cu_{10}Te_4S_{13}$ and its elemental distribution mapping. 7

Figure S3. The coordination modes of S for $Cu_{10}Te_4S_{13}$. 8

Figure S4. The experimental band gap of $Cu_{10}Te_4S_{13}$. 8

Figure S5. Electronic band structure of $Cu_{10}Te_4S_{13}$: (a) PBE without spin-orbit coupling, (b) PBE with spin-orbit coupling, and (c) HSE calculation. 9

Table S1. Crystal data and structure refinement for $Cu_{10}Te_4S_{13}$. 10

Table S2. Fractional atomic coordinates ($\times10^4$) and equivalent isotropic displacement parameters ($Å^2\times10^3$) for $Cu_{10}Te_4S_{13}$. 11

Table S3. The bond lengths (Å), calculated bond valence sums and global instability index(G) values for $Cu_{10}Te_4S_{13}$. 11

Table S4. The bond angles (deg.) for $Cu_{10}Te_4S_{13}$. 13

Table S5. Bond and atomic Mulliken population (MP) of $Cu_{10}Te_4S_{13}$ (CTS) and $AgGaS_2$ (AGS). 13

Table S6. A comparison of transparency range between $Cu_{10}Te_4S_{13}$ and some typical IR NLO materials. 14

Table S7. A summary of SHG effect and IR cutoff edge between $Cu_{10}Te_4S_{13}$ and some typical IR NLO materials, including those with stereochemically active lone pair (SCALP) units and containing Cu^+, with $AgGaS_2$ used as the reference..15

Experimental Section

Reagents

All the chemicals were obtained from commercial sources and used without further purification: CuCl (Admas-beta, 98%), Cu (Sinopharm Chemical Reagent, 99.99%), Te (Aladdin, 99.99%)), and S (Aladdin, 99.95 %). To prevent the possible oxidation of the metal and the potential deliquescence of the chloride, they are stored in a dry argon-filled glovebox.

Syntheses

Single crystalline samples of $Cu_{10}Te_4S_{13}$ were successfully synthesized via high-temperature solid-state reactions. The starting mixtures of CuCl, Cu, Te, and S, in stoichiometric ratios of 1: 2: 1: 3, were weighed and loaded into quartz crucible with an inner diameter of 7 mm. Following initial mixing, the samples were sealed in an 11 mm inner-diameter silicon tube using an oxyhydrogen flame under vacuum conditions (10^{-3} Pa). The sealed quartz tubes were then placed in a muffle furnace with a pre-programmed temperature profile, gradually heated to 823 K over a period of 10 hours, maintained at this temperature for 50 hours, and subsequently cooled to 603 K at a controlled rate of 1.5 K/h. The furnace was subsequently switched off, allowing the samples to naturally cool down to room temperature. Bulk single crystal samples with a black metallic luster were obtained after being repeatedly washed with hot water and dried, yielding an approximate crystallization efficiency of 60%.

Powder X-ray diffraction

Powder X-ray diffraction (PXRD) data of $Cu_{10}Te_4S_{13}$ were collected on the MiniFlex600 Rigaku X-ray Diffractometer using Cu Kα radiation (λ = 1.54186 Å) at room temperature in the angular range of 2θ = 5-70° with a scan step size of 0.02°.

Energy-dispersive X-ray spectroscopy

Microprobe elemental analysis was carried out with the aid of a field-emission scanning electron microscope (JSM6700F) outfitted with an energy-dispersive X-ray spectroscope (Oxford INCA).

Spectroscopic measurements

IR spectrum was carried out on a Magna 750 FT-IR spectrometer using air as background in the range of 4000–400 cm^{-1} with a resolution of 2 cm^{-1} at room temperature. The UV-vis-NIR spectrum was obtained in the range of 2000–200 nm by a PerkinElmer Lambda 900 spectrophotometer using $BaSO_4$ as the reference, and the reflection spectrum were converted into an absorption spectrum using the Kubelka-Munk function.[1] Absorption data was calculated from the diffuse reflection data by the Kubelka-Munk function: $\alpha/S = (1\text{-}R)^2/2R$, where α and S represent the absorption coefficient and the scattering coefficient, respectively. The band gap value can be given by extrapolating the absorption edge to the baseline in the α/S vs. energy graph.

Thermogravimetric analyses

Thermogravimetric analyses (TGA) were measured by NETZSCH STA 499C installation. The samples about 5.0–10.0 mg were placed in alumina crucibles and heated in 293-1293 K at a rate of 20 K/min under N_2 atmosphere.

Powder SHG measurements

The powder SHG property measurement was investigated by Kurtz-Perry method using a Q-switched laser radiation.[2] The laser radiation at 2900 nm was selected as the laser sources and $AgGaS_2$ were measured as the benchmark, respectively. Samples of $Cu_{10}Te_4S_{13}$ and $AgGaS_2$

were ground and sieved into different granule sizes (30−45, 45−75, 75−100, 100−150, and 150−210 μm) for the phase matching measurements. The SHG signals were detected via photomultiplier tube and oscilloscope.

Single-crystal X-ray diffraction

Single crystal X-ray diffraction data were obtained on the Rigaku Oxford X-ray diffractometer XtaLAB Synergy-R-Mo (λ = 0.71073 Å) at 293K. Data reduction and cell refinement and were performed with CrysAlisPro. The structure was solved by the direct methods and refined by full-matrix least-squares fitting on F^2 using *OLEX2-1.5* crystallographic software package.[3] All atoms were refined with anisotropic thermal parameters. The structural data were also checked by PLATON and no higher symmetry was found. The detailed crystallographic data for $Cu_{10}Te_4S_{13}$ were given in Table S1. The bond lengths, calculated bond valences and bond angles were listed in Table S3 and Table S4, respectively.

Computational Method

The DFT calculations have been performed using the *Vienna ab initio simulation package* (VASP)[4-6] with the Perdew-Burke-Ernzerhof (PBE)[7] exchange correlation functional. The projected augmented wave (PAW)[8] potentials with the valence states 4s, 4p and 3d for Cu, 5s and 5p for Te, 3s and 3p for S, respectively, have been used. A Γ-centered 7×7×5 Monkhorst-Pack grid for the Brillouin zone sampling[9] and a cutoff energy of 650 eV for the plane wave expansion were found to get convergent lattice parameters. The linear and nonlinear optical calculation was performed in the condition of a Monkhorst-Pack *k*-point mesh of 5×7×7. To avoid the underestimation of the semiconductor band gap inherent in PBE-based calculations,

we employed the Heyd-Scuseria-Ernzerhof (HSE06) screened hybrid density functional method to obtain the electronic structures. Additionally, the spin–orbit coupling (SOC) of Te^{4+} in $Cu_{10}Te_4S_{13}$ was included.

The imaginary part of the dielectric function due to direct inter-band transitions is given by the expression:

$$\varepsilon_2(\hbar\omega) = \frac{2e^2\pi}{\Omega\varepsilon_0}\sum_{k,v,c}\left|\langle\psi_k^c|u\cdot r|\psi_k^v\rangle\right|^2\delta(E_k^c - E_k^v - E)$$

where Ω, ω, u, v and c are the unit-cell volume, photon frequencies, the vector defining the polarization of the incident electric field, valence and conduction bands, respectively. The real part of the dielectric function is obtained from ε_2 by a Kramers-Kronig transformation:

$$\varepsilon_1(\omega) = 1 + \left(\frac{2}{\pi}\right)\int_0^{+\infty} d\omega' \frac{\omega'^2\varepsilon_2(\omega)}{\omega'^2 - \omega^2}$$

The refractive index $n(\omega)$ can be obtained based on ε_1 and ε_2.

In calculation of the static $\chi^{(2)}$ coefficients, the so-called length-gauge formalism[10] derived by Aversa and Sipe[11] and modified by Rashkeev et al[12] is adopted, which has proven to be successful in calculating the second order susceptibility for semiconductors and insulators. In the static case, the imaginary part of the static second-order optical susceptibility can be expressed as:

$$\chi^{abc} = \frac{e^3}{\hbar^2\Omega}\sum_{nml,k}\frac{r_{nm}^a\left(r_{ml}^b r_{ln}^c + r_{ml}^c r_{ln}^b\right)}{2\omega_{nm}\omega_{ml}\omega_{ln}}\left[\omega_n f_{ml} + \omega_m f_{ln} + \omega_l f_{nm}\right]$$
$$+ \frac{ie^3}{4\hbar^2\Omega}\sum_{nm,k}\frac{f_{nm}}{\omega_{mn}^2}\left[r_{nm}^a\left(r_{nm;c}^b + r_{mn;b}^c\right) + r_{nm}^b\left(r_{nm;c}^a + r_{mn;a}^c\right) + r_{nm}^c\left(r_{nm;b}^a + r_{mn;a}^b\right)\right]$$

where r is the position operator, $\hbar\omega_{nm} = \hbar\omega_n$ - $\hbar\omega_m$ is the energy difference for the bands m and n, $f_{mn} = f_m - f_n$ is the difference of the Fermi distribution functions, subscripts a, b, and c

are Cartesian indices, and $r^b_{mn;a}$ is the so-called generalized derivative of the coordinate operator in k space,

$$r^b_{nm;a} = \frac{r^a_{nm}\Delta^b_{mn} + r^b_{nm}\Delta^a_{mn}}{\omega_{nm}} + \frac{i}{\omega_{nm}} \times \sum_l (\omega_{lm} r^a_{nl} r^b_{lm} - \omega_{nl} r^b_{nl} r^a_{lm})$$

where $\Delta^a_{nm} = (p^a_{nn} - p^a_{mm}) / m$ is the difference between the electronic velocities at the bands n and m.

As the nonlinear optical coefficients is sensitive to the momentum matrix, much finer k-point grid and large amount of empty bands are required to obtain a convergent $\chi^{(2)}$ coefficient. The $\chi^{(2)}$ coefficients here were calculated from PBE wave functions and a scissor operator has been added to correct the conduction band energy (corrected to the experimental gap), which has proven to be reliable in predicting the second order susceptibility for semiconductors and insulators.

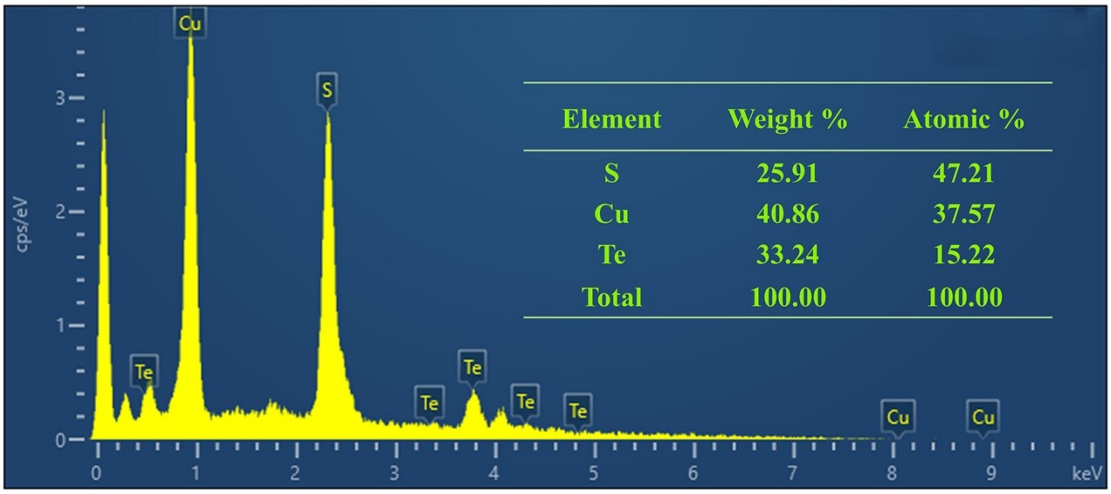

Figure S1. The energy-dispersive X-ray spectroscopy for $Cu_{10}Te_4S_{13}$.

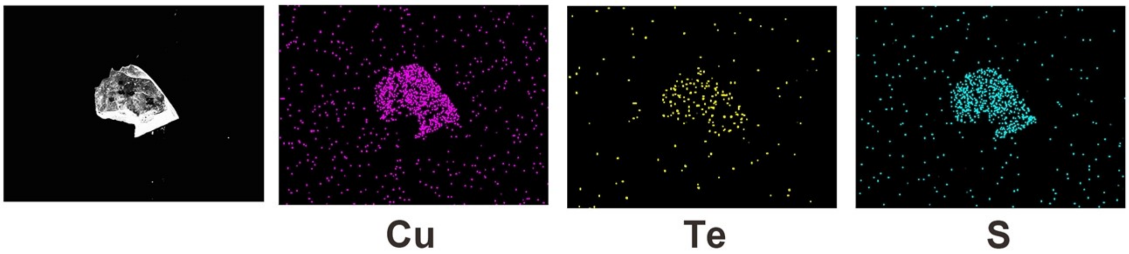

Figure S2. SEM images of $Cu_{10}Te_4S_{13}$ and its elemental distribution mapping.

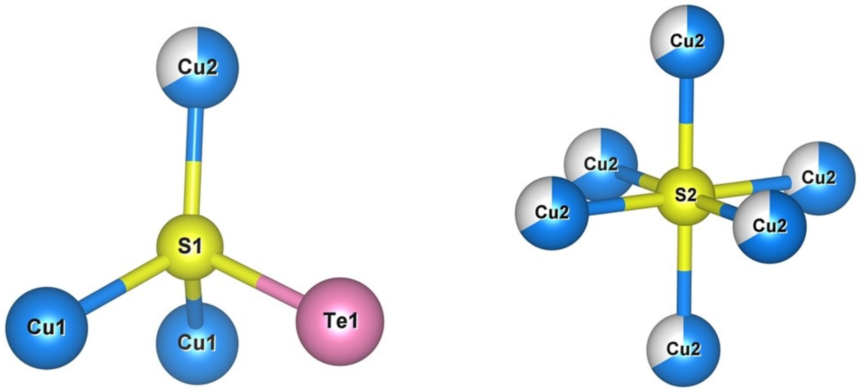

Figure S3. The coordination modes of S for $Cu_{10}Te_4S_{13}$.

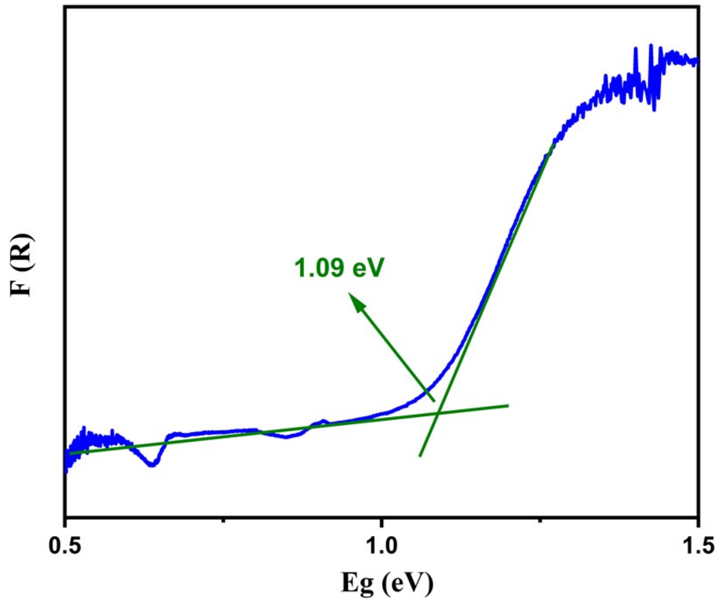

Figure S4. The experimental band gap of $Cu_{10}Te_4S_{13}$.

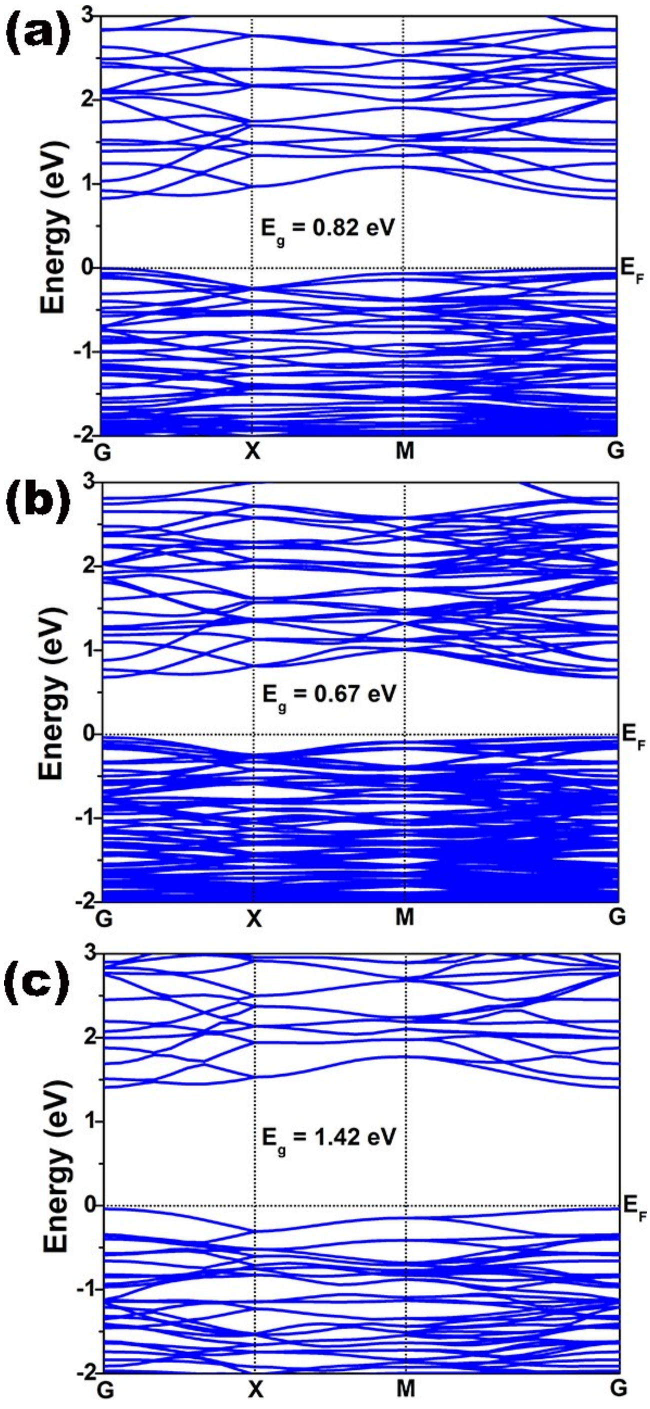

Figure S5. Electronic band structure of $Cu_{10}Te_4S_{13}$: (a) PBE without spin-orbit coupling, (b) PBE with spin-orbit coupling, and (c) HSE calculation.

Table S1. Crystal data and structure refinement for $Cu_{10}Te_4S_{13}$.

molecular formula	$Cu_{10}Te_4S_{13}$
Formula Weight	1562.58
crystal system	cubic
space group	$I\bar{4}3m$
Temperature(K)	293(2)
F(000)	1412.0
a/Å	10.26090(10)
b/Å	10.26090(10)
c/Å	10.26090(10)
α(deg)	90
β(deg)	90
γ(deg)	90
V/Å^3	1080.33(3)
Z	2
Dc(g.cm^{-3})	4.804
GOF on F^2	1.231
Flack factor	-0.002(16)
R_1,wR_2[I > 2σ(I)]$^\alpha$	0.0141, 0.0306
R1, wR_2 (all data)$^\alpha$	0.0141, 0.0306

$^\alpha$ $R_1 = \sum||F_o| - |F_c||/\sum|F_o|$, $wR_2 = \{\sum w[(F_o)^2 - (F_c)^2]^2/\sum w[(F_o)^2]^2\}^{1/2}$

Table S2. Fractional atomic coordinates (×10^4) and equivalent isotropic displacement parameters (Å^2×10^3) for $Cu_{10}Te_4S_{13}$.

	Atom	*Wyckoff*	x	y	z	U_{eq}(Å)
$Cu_{10}Te_4S_{13}$	Te1	8*c*	7603.4(3)	2396.6(3)	2396.6(3)	14.3(2)
	Cu1	12*d*	10000	2500	5000	24.2(4)
	Cu2	12*e*	7128(2)	5000	5000	28.7(4)
	S1	24*g*	8597.1(11)	3879.5(11)	3879.5(11)	15.2(4)
	S2	2*a*	5000	5000	5000	21.4(9)
	U_{eq} is defined as one third of the trace of the orthogonalized U_{ij} tensor.					

Table S3. The bond lengths (Å), calculated bond valence sums and global instability index(G) values for $Cu_{10}Te_4S_{13}$.

Compound	Atom	Bond	Bond-length	Bond-valence	BVS	G
$Cu_{10}Te_4S_{13}$	Cu1	Cu1-S1	2.3233(7)	0.2858	1.1432	0.20
		Cu1-S1#3	2.3233(7)	0.2858		
		Cu1-S1#4	2.3233(7)	0.2858		
		Cu1-S1#5	2.3233(8)	0.2858		
	Cu2	Cu2-S1	2.2173(18)	0.3807	1.1789	
		Cu2-S2	2.183(2)	0.4175		
		Cu2-S1#6	2.2173(18)	0.3807		
	Te	Te1-S1	2.3812(13)	1.2043	3.6129	
		Te1-S1#1	2.3812(13)	1.2043		
		Te1-S1#2	2.3812(13)	1.2043		
	S1	S1-Te1	2.3812(13)	0.2445	1.1968	
		S1-Cu1	2.3233(7)	0.2858		
		S1-Cu2	2.2173(18)	0.3807		
		S1-Cu1#7	2.3233(7)	0.2858		
	S2	S2-Cu2	2.183(2)	0.4175	2.5050	
		S2-Cu2#8	2.183(2)	0.4175		

		S2-Cu2#9	2.183(2)	0.4175		
		S2-Cu2#10	2.183(2)	0.4175		
		S2-Cu2#1	2.183(2)	0.4175		
		S2-Cu2#11	2.183(2)	0.4175		

Symmetry transformations used to generate equivalent atoms: #1 1-Y,+Z,1-X; #2 1-Z,1-X,+Y; #3 3/2-Y,1/2-Z,-1/2+X; #4 1/2+Y,1/2-Z,3/2-X; #5 2-X,+Y,1-Z; #6 +X,1-Y,1-Z;

Table S4. The bond angles (deg.) for $Cu_{10}Te_4S_{13}$.

$Cu_{10}Te_4S_{13}$			
S1#1-Te1-S1#2	97.98(5)	S1#4-Cu1-S1#5	111.79(3)
S1#1-Te1-S1	97.98(5)	S1#5-Cu1-S1	111.79(3)
S1#2-Te1-S1	97.98(5)	S1#4-Cu1-S1	104.93(6)
S1#3-Cu1-S1	111.79(3)	S1-Cu2-S1#6	94.33(12)
S1#4-Cu1-S1#3	111.79(3)	S2-Cu2-S1#6	132.83(6)
S1#5-Cu1-S1#3	104.93(6)	S2-Cu2-S1	132.83(6)

Symmetry transformations used to generate equivalent atoms: #1 1-Y,+Z,1-X; #2 1-Z,1-X,+Y; #3 3/2-Y,1/2-Z,-1/2+X; #4 1/2+Y,1/2-Z,3/2-X; #5 2-X,+Y,1-Z; #6 +X,1-Y,1-Z;

Table S5. Bond and atomic Mulliken population (MP) of $Cu_{10}Te_4S_{13}$ (CTS) and $AgGaS_2$ (AGS).

	Bond	MP	Length (Å)	Atom	MP	Charge (e)
CTS	Cu(1)-S(1)	0.38	2.32329	Cu(1)	10.91	0.09
	Cu(2)-S(1)	0.56	2.21723	Cu(2)	10.97	0.03
	Cu(2)-S(2)	0.30	2.18352	Te	5.14	0.86
	Te-S(1)	0.32	2.38120	S(1)	6.31	-0.31
				S(2)	6.46	-0.46
AGS	Ag-S	0.29	2.60513	Ag	18.98	0.02
	Ga-S	0.42	2.23581	Ga	12.18	0.82
				S	6.42	-0.42

Table S6. A comparison of transparency range between $Cu_{10}Te_4S_{13}$ and some typical IR NLO materials.

Compound	Space group	Transparency range (μm)	References
$AgGaS_2$	$I\bar{4}2d$	0.48–11.4	13
$AgGaSe_2$	$I\bar{4}2d$	0.76–17	13
$ZnGeP_2$	$I\bar{4}2d$	0.74–12	13
$AgGaTe_2$	$I\bar{4}2d$	0.91–23	14
$Sn_2Ga_2S_5$	$Pna2_1$	0.57–13.8	15
$BaGa_4S_7$	$Pmn2_1$	0.35–13.7	16
$[Ba_4Cl_2][ZnGa_4S_{10}]$	$I\bar{4}$	0.29–13.7	17
$[CsBa_3Cl_2][Ga_5S_{10}]$	$I\bar{4}$	0.31–12.5	18
$Nd_3[Ga_3O_3S_3][Ge_2O_7]$	$P\bar{6}2c$	0.25–13.7	19
Ag_2GeS_3	$Cmc2_1$	0.59–12.95	20
$Sr_2HgGe_2OS_6$	$P\bar{4}2_1m$	0.33–14.4	21
$SrCdGe_2OS_6$	$P\bar{4}2_1m$	0.34–12.0	22
$SrGeOSe_2$	$P2_12_12_1$	0.38–12.6	23
$SrCdSiS_4$	$Ama2$	0.33–18.2	24
Cd_4SiS_6	Cc	0.45–17.6	25
Cd_4SiSe_6	Cc	0.64–20.3	25
$CuHgPS_4$	$Pna2_1$	0.54–16.7	26
$CuZnPS_4$	$I\bar{4}2m$	0.41–16.5	27
$RbBiP_2S_6$	$P2_1$	0.5–15.0	28
$Hg_7P_2Se_{12}$	$P1$	0.84–22.8	29
$Ba_2SnSSi_2O_7$	$P4bm$	0.27–11.14	30
Hg_3AsS_4Cl	$P6_3mc$	0.52–13.7	31
Hg_3AsS_4Br	$P6_3mc$	0.51–14.2	31

**$Cu_{10}Te_4S_{13}$**	***$I\bar{4}3m$***	**0.84–25**	**this work**

Table S7. A summary of SHG effect and IR cutoff edge between $Cu_{10}Te_4S_{13}$ and some typical IR NLO materials, including those with stereochemically active lone pair (SCALP) units and containing Cu^+, with $AgGaS_2$ used as the reference.

Compound	Space group	SHG (×$AgGaS_2$)[a]	IR cutoff edge (μm)	References
$AgGaS_2$	$I\bar{4}2d$	1	11.4(S)[b]	13
$Ba_3(BS_3)(SbS_3)$	$P\bar{6}2m$	3	11(P)[c]	32
$Sn_2Ga_2S_5$	$Pna2_1$	0.8	13.8(S)	15
$RbPbPS_4$	$P2_12_12_1$	1.4	18.1(S)	33
Hg_3AsS_4Cl	$P6_3mc$	1.9	13.7(P)	31
Hg_3AsS_4Br	$P6_3mc$	2.6	14.2(P)	31
KCu_4AsS_4	$P2_1$	1.0	>25(P)	34
$RbCu_4AsS_4$	$P2_1$	0.9	>25(P)	34
$CsMnAs_3S_6$	$R3$	0.7	23.5(P)	35
$RbMnAs_3S_6$	$R3$	0.6	23.5(P)	35
$RbCuGa_6S_{10}$	Cc	1.5	20(P)	36
$CsCuGa_6S_{10}$	Cc	1.8	20(P)	36
$Cu_4ZnGe_2S_7$	$C2$	0.6	25(P)	37
$Cu_4CdGe_2S_7$	$C2$	0.05	25(P)	37
$CuZnPS_4$	$I\bar{4}2m$	3	16.5(S)	27
**$Cu_{10}Te_4S_{13}$**	***$I\bar{4}3m$***	**1.05**	**>25**(S)	**this work**

[a] This refers to data for particles in the size range of 70–110 μm;
[b] This refers to single crystal data;
[c] This refers to powder sample data.

References

1. P. Kubelka and F. Munk, *Z. Techn. Phys.*, 1931, **12**, 593−601.
2. S. K. Kurtz and T. T. Perry, *J. Appl. Phys.*, 1968, **39**, 3798–3813.
3. O. V. Dolomanov, L. J. Bourhis, R. J. Gildea, J. A. K. Howard and H. Puschmann, *J. Appl. Crystallogr.*, 2009, **42**, 339–341.
4. G. Kresse and D. Joubert, *Phys. Rev. B: Condens. Matter*, 1999, **59**, 1758–1775.
5. G. Kresse and J. Furthmüller, *Phys. Rev. B: Condens. Matter*, 1996, **54**, 11169–11186.
6. G. Kresse, http://cms.mpi.univie.ac.at/vasp/vasp/vasp.html.
7. J. P. Perdew, K. Burke and M. Ernzerhof, *Phys. Rev. Lett.*, 1996, **77**, 3865.
8. P. E. Blöchl, *Phys. Rev. Lett.*, 1994, **50**, 17953–17979.
9. D. J. Chadi, *Phys. Rev. Lett.*, 1977, **16**, 1746–1747.
10. Z. Fang, J. Lin, R. Liu, P. Liu, Y. Li, X. Huang, K. Ding, L. Ning and Y. Zhang, *CrystEngComm*, 2014, **16**, 10569–10580.
11. C. Aversa and J. E. Sipe, *Phys. Rev. Lett.*, 1995, **52**, 1570–1573.
12. S. N. Rashkeev, W. R. L. Lambrecht and P. B. Segall, *Phys. Rev. Lett.*, 1998, **57**, 3905–3919.
13. D. N. Nikogosyan, *Nonlinear Optical Crystals: A Complete Survey*, Springer, Berlin, 2005.
14. L. Bai, Z. Lin, Z. Wang, C. Chen and M.-H. Lee, *J. Chem. Phys.*, 2004, **120**, 8772–8778.
15. M.-Y. Li, B. Li, H. Lin, Z. Ma, L.-M. Wu, X.-T. Wu and Q.-L. Zhu, *Chem. Mater.*, 2019, **31**, 6268–6275.
16. X. Lin, G. Zhang and N. Ye, *Cryst. Growth Des.*, 2009, **9**, 1186–1189.

17. H. Chen, Y.-Y. Li, B. Li, P.-F. Liu, H. Lin, Q.-L. Zhu and X.-T. Wu, *Chem. Mater.*, 2020, **32**, 8012–8019.

18. B.-W. Liu, H.-Y. Zeng, X.-M. Jiang and G.-C. Guo, *CCS Chemistry*, 2021, **3**, 964–973.

19. M. Y. Ran, S. H. Zhou, W. B. Wei, B. X. Li, X. T. Wu, H. Lin and Q. L. Zhu, *Small*, 2023, **19**, 2300248.

20. H. Chen, M. Y. Ran, S. H. Zhou, X. T. Wu and H. Lin, *Adv. Optical Mater.*, 2024, **12**, 2401100.

21. M.-Y. Ran, S.-H. Zhou, X.-T. Wu, H. Lin and Q.-L. Zhu, *Mater. Today Phys.*, 2024, **44**, 101442.

22. M. Y. Ran, S. H. Zhou, B. X. Li, W. B. Wei, X. T. Wu, H. Lin and Q. L. Zhu, *Chem. Mater.*, 2022, **34**, 3853–3861.

23. M.-Y. Ran, Z. Ma, H. Chen, B. Li, X.-T. Wu, H. Lin and Q.-L. Zhu, *Chem. Mater.*, 2020, **32**, 5890–5896.

24. H. D. Yang, M. Y. Ran, S. H. Zhou, X. T. Wu, H. Lin and Q. L. Zhu, *Chem. Sci.*, 2022, **13**, 10725–10733.

25. J. Chen, C. Lin, S. Yang, X. Jiang, S. Shi, Y. Sun, B. Li, S. Fang and N. Ye, *Cryst. Growth Des.*, 2020, **20**, 2489–2496.

26. M.-Y. Li, Z. Ma, B. Li, X.-T. Wu, H. Lin and Q.-L. Zhu, *Chem. Mater.*, 2020, **32**, 4331–4339.

27. Z. Li, S. Zhang, Z. Huang, L.-D. Zhao, E. Uykur, W. Xing, Z. Lin, J. Yao and Y. Wu, *Chem. Mater.*, 2020, **32**, 3288–3296.

28. M.-M. Chen, S.-H. Zhou, W. Wei, M.-Y. Ran, B. Li, X.-T. Wu, H. Lin and Q.-L. Zhu,

ACS Materials Lett., 2022, **4**, 1264–1269.

29. Y. Chu, H. Wang, Q. Chen, X. Su, Z. Chen, Z. Yang, J. Li and S. Pan, *Adv. Funct. Mater.*, 2023, **34**, 2314933.
30. Y.-F. Shi, Z. Ma, B.-X. Li, X.-T. Wu, H. Lin and Q.-L. Zhu, *Mater. Chem. Front.*, 2022, **6**, 3054–3061.
31. F. Xu, X. Xu, B.-X. Li, G. Zhang, C. Zheng, J. Chen and N. Ye, *Inorg. Chem. Front.*, 2024, **11**, 2105–2115.
32. Y.-Y. Li, B.-X. Li, G. Zhang, L.-J. Zhou, H. Lin, J.-N. Shen, C.-Y. Zhang, L. Chen and L.-M. Wu, *Inorg. Chem.*, 2015, **54**, 4761–4767.
33. A. Y. Wang, S.-H. Zhou, M.-Y. Ran, B. Li, X.-T. Wu, H. Lin and Q.-L. Zhu, *Inorg. Chem. Front.*, 2024, **11**, 3744–3754.
34. X. Zhao, C. Lin, C. Wang, H. Tian, P. Dong, T. Yan, B. Li, N. Ye and M. Luo, *Scr. Mater.*, 2024, **242**, 115935.
35. R. Ye, B. W. Liu, X. M. Jiang, J. Lu, H. Y. Zeng and G. C. Guo, *ACS Appl. Mater. Interfaces*, 2020, **12**, 53950–53956.
36. K. Li, X. Zhang, B. Chai, H. Yu, Z. Hu, J. Wang, Y. Wu and H. Wu, *Chem. Eur. J.*, 2024, e202403515, DOI: 10.1002/chem.202403515.
37. J. C. Kelly, C. E. Hoyer, S. H. Shin, J. Bin Cho, S. S. Stoyko, A. Bonnoni, A. J. Craig, K. E. Rosello, E. T. O'Hara, J. I. Jang and J. A. Aitken, *J. Alloys Compd.*, 2023, **970**, 172437.
